# Vasohibin-1 as a novel microenvironmental biomarker for patient risk reclassification in low-risk prostate cancer

**DOI:** 10.18632/oncotarget.23011

**Published:** 2017-12-07

**Authors:** Hiroaki Kobayashi, Takeo Kosaka, Shuji Mikami, Yasumasa Miyazaki, Kazuhiro Matsumoto, Eiji Kikuchi, Akira Miyajima, Kaori Kameyama, Yasufumi Sato, Mototsugu Oya

**Affiliations:** ^1^ Department of Urology, Keio University School of Medicine, Tokyo, Japan; ^2^ Division of Diagnostic Pathology, Keio University School of Medicine, Tokyo, Japan; ^3^ Department of Vascular Biology, Institute of Development, Aging and Cancer, Tohoku University, Sendai, Japan

**Keywords:** prostate cancer, vasohibin, prostatic biopsy, angiogenesis, immunohistochemistry

## Abstract

**Background:**

We previously reported high expression of vasohibin-1 (VASH1), which is specifically expressed in activated vascular endothelial cells, was a prognostic indicator of disease progression in prostate cancer. The aim of this study was to assess whether VASH1 expression at the area of normal prostatic tissue as well as that of intratumoral tissue could reflect the grade of malignancy of prostate cancer.

**Results:**

Pathological upgrade of Gleason Score ≥7 by radical prostatectomy was observed in 48 patients (upgraded group). The median VASH1 densities of the intratumoral and normal areas were 9.7 ± 9.5 and 13.3 ± 11.8, respectively, and the median MVDs were 58.6 ± 20.3 and 64.1 ± 23.5, respectively. We detected a strong positive correlation with each other for both VASH1 density (*ρ* = 0.589, *p* < 0.001) and MVD (*ρ* = 0.342, *p* < 0.001). VASH1 density was significantly higher in the upgreaded group than in the non-upgraded group regardless of prostatic location (intratumoral area: *p* < 0.001, normal area: *p* < 0.001).

**Conclusions:**

Even if the tumor volume was low in biopsy samples, VASH1 density reflected the grade of malignancy throughout the prostate. These results suggested that VASH1 expression could be a novel microenvironmental biomarker for patient risk reclassification in low-risk prostate cancer.

**Materials and Methods:**

Among the 1177 patients who underwent radical prostatectomy, 104 patients diagnosed with Gleason Score ≤6 and positive cores ≤3 were included. We immunohistochemically examined the microvessels positive for anti-CD34 as microvessel density (MVD), and those with activated endothelial cells as VASH1 density using prostatic biopsy samples, and evaluated the association between their expressions and clinicopathological findings.

## INTRODUCTION

Prostate cancer (PCa) is one of the most common types of cancer in men worldwide and its incidence is still increasing due to prostate-specific antigen (PSA) screening, the development of diagnostic imaging system, and innovative ideas of biopsy methods [1–3]. These advancements of diagnostic performance might bring about overdiagnosis by detecting insignificant PCa that do not require immediate treatment [4]. Thus, nowadays, active surveillance is an accepted management strategy for low-risk PCa, as most of these patients are unlikely to die of PCa [5]. However, this strategy has some indeterminate factors with risk classification, inclusion criteria, and the follow-up schedule that are still based on arbitrary recommendations rather than high-level evidence. Therefore, novel biomarkers that are expressed throughout the prostate, and reflect the malignant potential for high grade and/or invasiveness are highly required.

Angiogenesis, that is the growth of new blood vessels, not only plays a role in human normal development but also in pathophysiological conditions such as inflammation and cancer [6]. Increased vascularity through angiogenesis enhances growth of the primary tumor by supplying nutrients and oxygen, and provides an avenue for hematogenous metastasis [7–9]. One of the most commonly used techniques to quantify intratumoral angiogenesis is microvessel density (MVD) assessment [10]. Some studies indicated that MVD served as a predictor of poorly differentiated PCa and biochemical PSA failure after treatment [11–13]. However, to date evidence of the prognostic role of MVD is contradictory because MVD corresponds to the number of accomplished vessels, including the quiescent vessels. Thus, MVD cannot reflect angiogenic activity alone.

We recently isolated a novel endothelium-derived negative feedback inhibitor or suppressor of angiogenesis, vasohibin-1 (VASH1), which is an intrinsic factor specifically expressed in activated vascular endothelial cells (ECs). VASH1 has been induced by representative angiogenic factors, such as vascular endothelial growth factor (VEGF) and fibroblast growth factor 2 (FGF-2), through PKCδ [14]. Previous studies found that the expression of VASH1 was restricted to ECs of blood vessels in the tumor stroma, and correlated with the expression of VEGF and FGF-2 in tumor cells [15]. Based on these findings, we also previously reported that high VASH1 expression was a prognostic indicator of disease progression and could serve as a novel biomarker for predicting PCa progression [16].

In the present study, we examined the expression of VASH1 density and MVD using the transrectal needle biopsy (TRNBx) samples in patients with clinically low-risk PCa. The aim of this study was to determine the strength of a correlation of VASH1 expression as tumor microenvironment between the area of intratumoral and normal tissue, and to assess whether VASH1 expression could reflect the grade of malignancy throughout the prostate.

## RESULTS

### Patient characteristics in low-risk PCa

The patient characteristics and correlation of clinicopathological parameters and VASH1 density or MVD of 104 patients are shown in Table 1. The median age of all patients was 65 ± 5.7 years and median PSA level at diagnosis was 6.1 ± 4.4 ng/ml. Pathological upgrade for GS *≥*7 by RP was observed in 48 (46.2%) patients and stage progression ≥pT3a was observed in 7 (6.7%) patients. During a median follow-up of 70.2 months, only 7 (6.7%) patients experienced subsequent biochemical PSA recurrence and no one died of PCa.

**Table 1:** Correlation of clinicopathological parameters and VASH1 density or MVD in 104 patients

		VASH1 density				MVD			
	Number of patients	Intratumoral tissue area (mean ± SD)	*P*-value	Normal tissue area (mean ± SD)	*P*-value	Intratumoral tissue area (mean ± SD)	*P*-value	Normal tissue area (mean ± SD)	*P*-value
Total	104	9.7 ± 9.5		13.3 ± 11.8		58.6 ± 20.3		64.1 ± 23.5	
Age (years)									
≤65	57 (54.8%)	9.1 ± 10.1	0.293	11.7 ± 11.5	0.127	57.0 ± 19.7	0.365	64.0 ± 23.4	0.919
>65	47 (45.2%)	10.4 ± 8.7		15.1 ± 12.0		60.6 ± 21.0		64.3 ± 23.9	
PSA at diagnosis									
≤10.0	86 (82.7%)	9.7 ± 9.4	0.896	13.0 ± 11.4	0.779	58.3 ± 20.0	0.780	64.9 ± 24.6	0.542
>10.0	18 (17.3%)	9.6 ± 10.1		14.6 ± 13.6		60.2 ± 21.9		60.5 ± 17.5	
Prostate estimation									
≤30.0	31 (29.8%)	9.3 ± 9.9	0.660	11.5 ± 12.6	0.174	59.0 ± 22.3	0.998	62.2 ± 25.7	0.334
>30.0	73 (70.2%)	9.9 ± 9.4		14.0 ± 11.4		58.4 ± 19.5		64.9 ± 22.7	
cT stage									
cT1c	35 (33.7%)	7.7 ± 6.8	0.310	11.6 ± 9.9	0.493	51.9 ± 14.1	**0.046**	61.7 ± 25.3	0.246
cT2	69 (66.3%)	10.8 ± 10.5		14.1 ± 12.6		62.0 ± 22.1		64.9 ± 22.7	
Gleason Score (RP)									
≤6	56 (53.8%)	7.1 ± 8.0	**0.003**	9.5 ± 10.9	**<0.001**	57.2 ± 19.4	0.432	61.8 ± 22.0	0.417
≥7	48 (46.2%)	12.7 ± 10.3		17.7 ± 11.4		60.3 ± 21.3		66.8 ± 25.1	
pT stage									
≤T2	97 (93.3%)	9.6 ± 9.5	0.701	12.8 ± 11.6	0.434	58.7 ± 20.4	0.491	64.2 ± 23.8	0.519
≥T3	7 (6.7%)	11.8 ± 10.1		19.4 ± 13.2		57.2 ± 19.3		62.9 ± 19.8	
Tumor multiplicity									
Solitary	38 (36.5%)	7.6 ± 8.1	0.116	9.7 ± 11.5	**0.004**	56.6 ± 18.3	0.568	61.3 ± 23.4	0.287
Multiple	66 (63.5%)	10.9 ± 10.1		15.3 ± 11.5		59.8 ± 21.4		65.8 ± 23.6	
PSA recurrence									
No	97 (93.3%)	9.8 ± 9.6	0.772	13.3 ± 11.8	0.990	58.1 ± 20.5	0.217	63.4 ± 24.0	0.109
Yes	7 (6.7%)	8.6 ± 8.7		12.9 ± 12.0		65.5 ± 17.6		73.6 ± 12.4	

Abbreviations: RP; radical prostatectomy, VASH1; vasohibin-1, MVD; microvessel density.

### Evaluation of the difference of VASH1 density and MVD according to prostatic location, and correlation of each clinicopathological feature

**Figure 1:**
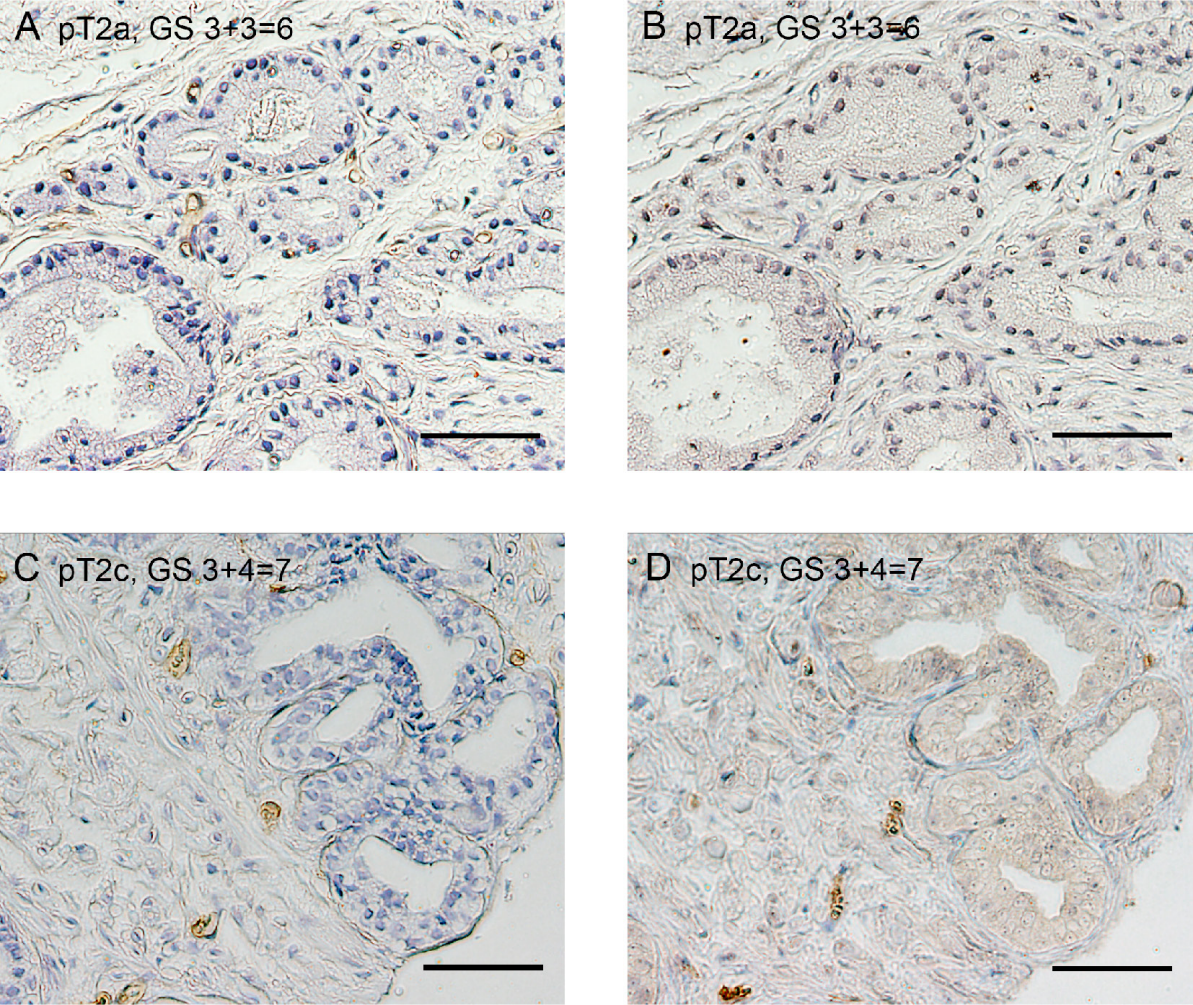
Immunostaining for CD34 (**A** and **C**) and VASH1 (**B** and **D**) at the intratumoral area in patients with low-risk PCa. Low VASH1 density (A and B) and high VASH1 density (C and D). Bar = 0.1 mm.

**Figure 2:**
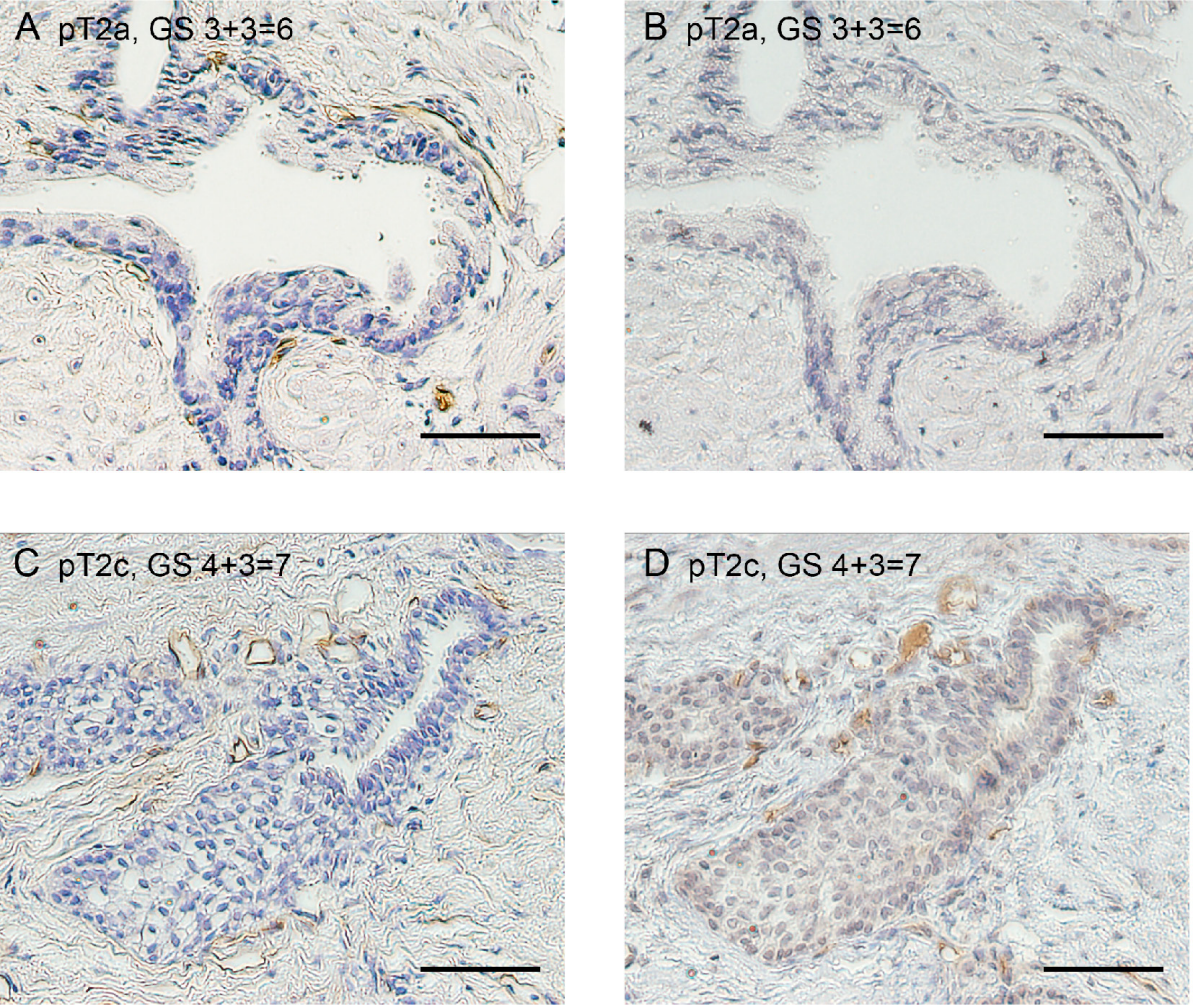
Immunostaining for CD34 (**A** and **C**) and VASH1 (**B** and **D**) at the normal area in patients with low-risk PCa. Low VASH1 density (A and B) and high VASH1 density (C and D). Bar = 0.1 mm.

We examined the immunohistochemical expression of VASH1 and CD34 at both of the intratumoral and normal areas to elucidate the biological significance of VASH1 in clinically low-risk PCa (Figures 1, 2). VASH1 staining of vascular ECs was negative or negligible in GS *≤*6 cancer (Figures 1B, 2B) while strong VASH1 staining was observed in GS ≥7 cancer in over half of the cases (Figures 1D, 2D).

The median VASH1 density (counts per mm^2^) at the intratumoral and normal areas were 9.7 ± 9.5 and 13.3 ± 11.8, respectively (Table 1). The patients with GS *≥*7 by RP had a higher VASH1 density than those with GS ≤6 at both the intratumoral (*p* < 0.001) and normal areas (*p* < 0.001). The level of VASH1 density at the normal area was significantly higher in multiple tumors than solitary tumors (*p* = 0.004). Other clinicopathological factors such as PSA level at diagnosis, clinical and pathological T stage were not significantly associated with VASH1 density. Meanwhile, the median MVD (counts per mm^2^) at the intratumoral and normal areas were 58.6 ± 20.3 and 64.1 ± 23.5, respectively. There were no significant differences between MVD and clinicopathological factors such as GS by RP, tumor multiplicity, PSA level at diagnosis, and pT stage regardless of prostatic location.

As it has been reported that VASH1 associates with CD34, we also investigated the relationship between VASH1 and CD34 expression. Using the Spearman correlation coefficient test, we detected a significant positive correlation between VASH1 density and MVD at the intratumoral area (*ρ* = 0.323, *p* = 0.001) and at the normal area (*ρ* = 0.444, *p* < 0.001). Moreover, we examined the relationship of VASH1 density and MVD according to the prostatic location focusing on tumor heterogeneity. We detected a strong positive correlation between the intratumoral and normal areas in both VASH1 density (*ρ* = 0.589, *p* < 0.001) and MVD (*ρ* = 0.342, *p* < 0.001).

### Clinical value of VASH1 density and MVD in low-risk PCa patients

We defined the “upgraded group” as the patients whose grade was pathologically upgraded for GS *≥*7, in other words unfavorable pathologic findings, and the “non-upgraded group” as not upgraded for GS *≤*6 when we divided the patients according to GS by RP. Clinicopathological differences of the two groups are shown in Table 2. The median values of VASH1 density and MVD were 8.3 and 54.9 per mm^2^, respectively. We used a median VASH1 density of ≥10 per mm^2^ and a MVD of *≥*55 per mm^2^ as the cutoff levels. There were no significant differences in patient age, PSA level and MVD at any location. Meanwhile, the upgraded group was significantly higher in cT stage (*p* = 0.032), pT stage (*p* = 0.003), and VASH1 density at both the intratumoral (*p* = 0.002) and normal areas (*p* = 0.001) than the non-upgraded group.

**Table 2:** Clinicopathological characteristics in 104 patients according to the presence of pathological upgrade

	Total	Upgraded group	Non-upgraded group	*P*-value
No. of patients	104	48	56	
Median age (range)	65 (49–75)	65 (50–74)	65 (49–75)	
≤65	57	27	30	0.784
>65	47	21	26	
Median PSA at diagnosis (range)	6.1 (3.0–24.9)	6.0 (3.0–24.8)	6.9 (3.5–24.9)	
≤10.0	86	41	45	0.497
>10.0	18	7	11	
cT stage				
cT1c	35	11	24	**0.032**
cT2	69	37	32	
pT stage				
≤pT2	97	41	56	**0.003**
≥pT3	7	7	0	
MVD at the intratumoral area				
<55	57	24	33	0.362
≥55	47	24	23	
VASH1 density at the intratumoral area				
<10	60	20	40	**0.002**
≥10	44	28	16	
MVD at the normal tissue area				
<55	41	17	24	0.439
≥55	63	31	32	
VASH1 density at the normal tissue area				
<10	49	14	35	**0.001**
≥10	55	34	21	

Abbreviations: VASH1; vasohibin-1, MVD; microvessel density.

## DISCUSSION

In the present study, we retrospectively examined the VASH1 and CD34 expression of intratumoral vessels of 104 low-risk PCa patients by immunohistochemical staining using TRNBx and compared it with those of normal prostatic tissue. Although there was a strong positive correlation between the two in both VASH1 density and MVD, VASH1 density was only significantly higher in the upgraded group than that in the non-upgraded group, not only at the intratumoral area but also at the normal area. Meanwhile, MVD had no significant differences.

Current NCCN guidelines and D' Amico classification define the low-risk PCa as clinical T1c-2a, GS *≤*6, and PSA <10 ng/ml [17, 18]. In addition, NCCN guidelines set up very low-risk PCa as clinical T1c, GS *≤*6, PSA <10 ng/ml, number of positive core <3, tumor content per positive biopsy core *≤*50%, and PSA density <0.15 ng/ml/g [17]. Thus, very low-risk PCa emphasized the pathological findings of prostatic biopsy more than low-risk PCa. Even low-risk PCa does not mean complete absence of risk for disease progression and cancer death. However, the majority of newly diagnosed low-risk PCa would undergo definitive therapy, despite the attendant long-term side effects and cost [19, 20]. A major drawback in the selection of appropriate treatment strategies of low-risk PCa is underestimation of tumor grade or stage at diagnosis. For instance, each positive biopsy core represents only some of the entire prostate, so another high grade or localized invasive PCa may remain elsewhere.

We hypothesized that the key points to calculate the grade of malignancy throughout the prostate was tumor microenvironment, and focused attention on angiogenesis that had a critical role in tumor growth and metastasis. One of the biomarkers that could reflect angiogenic aggressiveness as tumor microenvironment was MVD [10, 13]. Several studies on PCa indicated that the status of MVD was associated with GS and pathological stage, and could be a prognostic factor of patient survival [10, 12, 13, 21]. However, we found no significant association between MVD and pathological upgrade or stage progression in this study. One of the reasons might be because MVD corresponds to the number of accomplished vessels and includes vessels without the potential of neovascularization in tumors.

VASH1 has been isolated from VEGF inducible genes in ECs present in newly formed blood vessels behind the sprouting front where angiogenesis terminates [22, 23]. We previously reported that VASH1 was specifically expressed in activated vascular ECs and was associated with tumor malignancy such as GS, pT stage, and was an independent predictor of tumor progression in PCa [16]. Interestingly, that induction of VASH1 disappears under a hypoxic condition or in the presence of inflammatory cytokines [24]. These previous results strongly suggest that the status of VASH1 density could serve as an index of the malignant potential of tumor angiogenesis, and also the level of VASH1 expression might influence unfavorable pathologic findings [16, 25].

Indeed, level of VASH1 expression was higher in the upgraded group than the non-upgraded group and had a strong positive correlation with normal prostatic tissue. The results of this study suggest that since high GS tumors caused hyperactivity of angiogenesis throughout the prostate, negative cores of TRNBx as well as positive cores might be useful to examine for the grade of tumor malignancy. On the other hand, it has a possibility to miss a vital essentiality of cancer if we pay attention to only detected tumors. The results that VASH1 expression at normal tissue areas in multiple tumors was higher than in solitary tumors, indicated that hyperactivity of angiogenesis might represent the environment of occurrence of the de novo cancer more often anywhere.

Several limitations of our study should be considered. First and foremost, it was performed in a retrospective manner with a limited number of patients. Another limitation was that we did not perform MRI for all cohorts, which could have affected the diagnostic performance of PCa.

## CONCLUSIONS

Our results demonstrate that high VASH1 expression in positive cores as well as negative cores reflected unfavorable pathological findings in clinically low-risk PCa. These results indicate that VASH1 expression could be a novel microenvironmental biomarker for patient risk reclassification in localized PCa.

## MATERIALS AND METHODS

### Patient selection

We retrospectively analyzed the clinical records of 1177 patients who underwent laparoscopic radical prostatectomy (RP) at Keio University Hospital between January 2003 and January 2015. Among them, 104 patients who underwent TRNBx at our own hospital and were diagnosed with localized PCa with a Gleason score (GS) of *≤*6, total positive cores ≤3, and *≤*50% tumor content per positive biopsy core were included. None of the patients had received neoadjuvant hormonal treatment before RP. This retrospective clinical study was approved by the ethical committee of Keio University Hospital. The clinical characteristics of all cohorts are shown in Table 1. Clinical T stage was assessed by digital rectal examination (DRE) and magnetic resonance imaging (MRI), and cT2 was defined when the tumor was palpable or visible on imaging while cT1c was defined as negative DRE and the absence of suspicious lesions by MRI. Patients were followed by serum PSA level and imaging studies after RP. Biochemical PSA recurrence was defined by an elevation of serum PSA level at three consecutive measurements.

### Tissue samples and immunohistochemistry

All pathological specimens were re-reviewed by dedicated uropathologists to unify the reproducibility of the diagnosis. As for the pathologic stage, all tumors were classified according to the 2006 TNM staging system. We carried out immunohistochemical staining for CD34 (as a marker of vascular ECs) and VASH1. All specimens were fixed in 10% formalin, embedded in paraffin, and cut into 4 μm thick sections and placed on silane-coated glass slides. Tissue sections were deparafinized in xylene, and hydrated by immersion in graded alcohols and finally in distilled water. After antigen retrieval was performed, endogenous peroxidase activity was blocked by 0.3% hydrogen peroxidase. The tissue sections were then incubated with a blocking solution of 6% dry milk in PBS. The primary antibodies were all mouse monoclonal antibodies (mAbs): anti-human VASH1 mAb diluted at a concentration of 4 μg/ml and anti-CD34 (Nichirei Biosciences, Tokyo, Japan).

We previously described a mouse mAb against a synthetic peptide corresponding to the 286 to 299 amino acid sequence of VASH1 [25]. After washing with PBS, the tissue sections were incubated with secondary antibodies (Histofine Simple Stain MAX PO (M); Nichirei Biosciences). Color was developed with 3, 3′-diaminobenzamine tetrahydrochloride in 50 mmol/L Tris-HCl (pH 7.5) containing 0.005% hydrogen peroxide. The sections were counterstained with haematoxylin. The positive control slide CD34 antigen was prepared from paraffin-fixed bladder cancer tissue with high MVD. The appropriate negative controls slides for CD34 antigen and VASH1 were prepared by substituting the primary antibody with the immune globulin fraction of nonimmune mouse serum at the same concentration in each staining run.

### Evaluation of immunostaining

**Figure 3:**
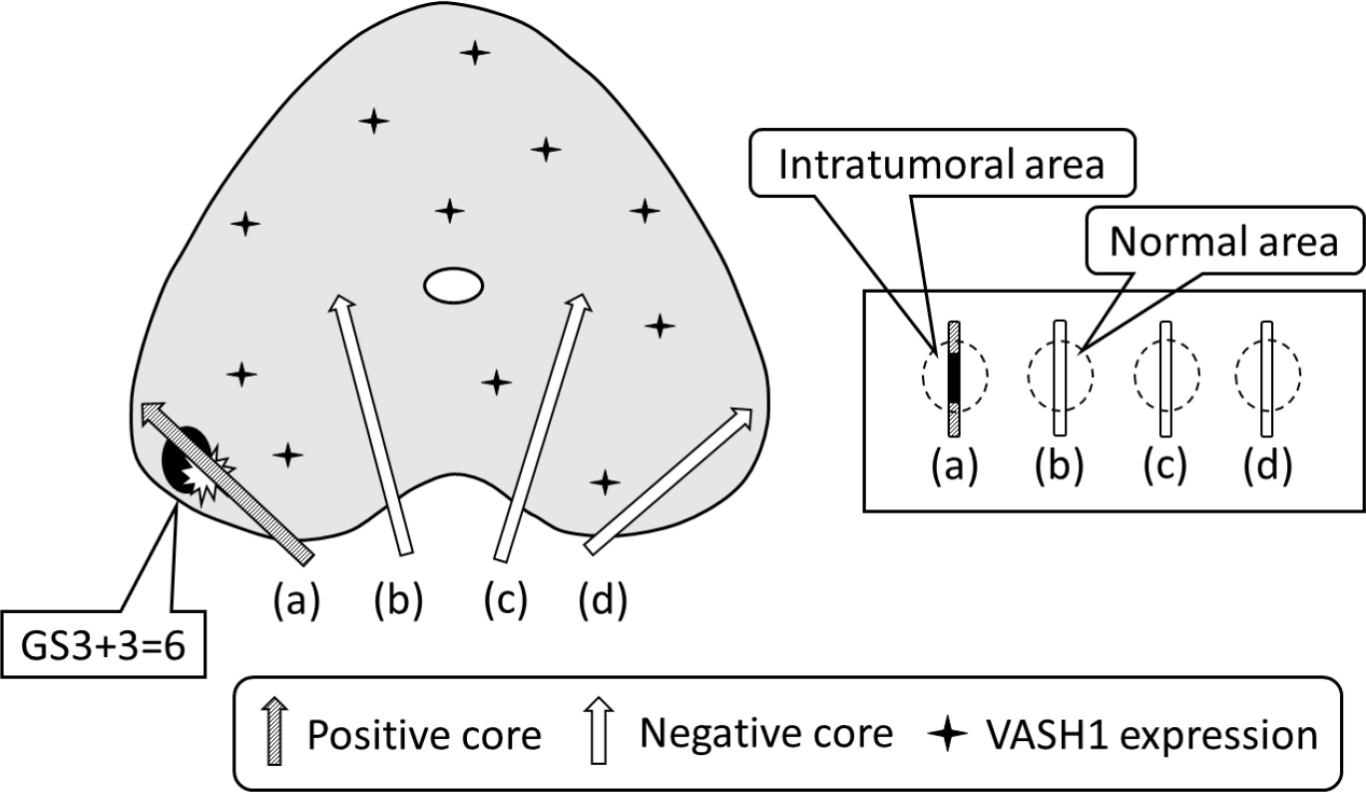
The total image of prostate cancer with tumor microenvironment and definition of the “intratumoral area” and “normal area”

Two authors independently evaluated immunoreactivity. They were blinded to the clinical course of the patients and the average of the numbers counted by the 2 investigators was used for subsequent analyses. Olympus IX71 (Olymus, Tokyo, Japan) was used for the analysis. We counted the microvessels at the neighborhood of cancer in positive cores as the “intratumoral area” and at the normal tissue which included the largest possible number of ductuli separately in negative cores as the “normal area” (Figure 3). Microvessels were identified on the basis of their architecture, lumen lined by ECs, complemented by positivity of the ECs for anti-CD34 after scanning the immunostained section at low magnification (×40 and ×100). The areas with the highest number of distinctly highlighted microvessels were selected and counted at high magnification (×200). Any immunostained EC or cluster separated from adjacent vessels was counted as a single microvessel, even in the absence of vessel lumen. Each single count was defined as the highest number of microvessels identified at the “hot spot” as shown previously [13, 26–28]. The highest number of microvessels in the hot spot was counted for MVD. VASH1-positive signals were counted in the “hot spot” in which the highest number of vessels positive for anti-CD34 was identified. We regarded the number of VASH1-positive signals per mm^2^ as “VASH1 density” [28–30].

### Statistical analysis

The associations between each clinicopathological parameter and VASH1 density or MVD of each location were validated using χ^2^ test or Mann-Whitney *U*-test. Differences among groups were regarded as significant when *P* < 0.05. These analyses were conducted with the SPSS version 22.0 statistical software package.

## Abbreviations

PCa: prostate cancer
PSA: prostate-specific antigen
MVD: microvessel density
ECs: endothelial cells
VASH1: vasohibin-1
VEGF: vascular endothelial growth factor
FGF-2: fibroblast growth factor 2
TRNBx: transrectal needle biopsy
RP: radical prostatectomy
GS: Gleason score
DRE: digital rectal examination
MRI: magnetic resonance imaging
mAbs: monoclonal antibodies
